# The impact of gastric acid suppressants on peritonitis risk in peritoneal dialysis patients: a systematic review and meta-analysis

**DOI:** 10.1093/ckj/sfaf054

**Published:** 2025-02-20

**Authors:** Ching-Chung Hsiao, Chieh-Li Yen, Shu-Chun Huang, Cheng-Yi Chen, Ya-Chung Tian, Yung-Chang Chen, Wen-Yu Ho, Jia-Jin Chen

**Affiliations:** Department of Nephrology, New Taipei City Municipal Tucheng Hospital, New Taipei City, Taiwan; Chang Gung University College of Medicine, Taoyuan City, Taiwan; Chang Gung University College of Medicine, Taoyuan City, Taiwan; Department of Nephrology, Linkou Chang Gung Memorial Hospital, Taoyuan City, Taiwan; Kidney Research Center, Linkou Chang Gung Memorial Hospital, Taoyuan City, Taiwan; Department of Physical Medicine and Rehabilitation, New Taipei Municipal TuCheng Hospital, New Taipei City, Taiwan; Division of Nephrology, Department of Internal Medicine, Mackay Memorial Hospital Hsinchu, Hsinchu, Taiwan; Chang Gung University College of Medicine, Taoyuan City, Taiwan; Department of Nephrology, Linkou Chang Gung Memorial Hospital, Taoyuan City, Taiwan; Kidney Research Center, Linkou Chang Gung Memorial Hospital, Taoyuan City, Taiwan; Chang Gung University College of Medicine, Taoyuan City, Taiwan; Department of Nephrology, Linkou Chang Gung Memorial Hospital, Taoyuan City, Taiwan; Kidney Research Center, Linkou Chang Gung Memorial Hospital, Taoyuan City, Taiwan; Chang Gung University College of Medicine, Taoyuan City, Taiwan; Department of Nephrology, Linkou Chang Gung Memorial Hospital, Taoyuan City, Taiwan; Kidney Research Center, Linkou Chang Gung Memorial Hospital, Taoyuan City, Taiwan; Chang Gung University College of Medicine, Taoyuan City, Taiwan; Department of Nephrology, Linkou Chang Gung Memorial Hospital, Taoyuan City, Taiwan; Kidney Research Center, Linkou Chang Gung Memorial Hospital, Taoyuan City, Taiwan

To the Editor,

The 2022 International Society for Peritoneal Dialysis (ISPD) peritonitis guidelines [1] suggest that ‘avoiding or limiting the use of histamine-2 receptor antagonists (H2RAs) may prevent enteric peritonitis in peritoneal dialysis (PD) patients.’ Recently, a meta-analysis by Yao *et al*. claims a significant association between gastrointestinal acid suppressant (GAS) use and enteric peritonitis [odds ratio (OR) 1.61, 95% confidence interval (CI) 1.26–2.05] [2]. While their findings align with the ISPD recommendation, we raise some methodological concerns and present results from our meta-analysis, which provide a contrasting perspective.

One critical issue with Yao *et al*.’s study is the inclusion of two studies (Gabella *et al*. [S1] and Nessim *et al*. [S2]) that compared GAS use between enteric and non-enteric peritonitis PD patients but lacked a non-peritonitis control group. Without such a control, it is impossible to determine whether GAS exposure independently contributes to the development of peritonitis. Another major limitation is the conflation of overall PD-associated peritonitis and enteric peritonitis, which are distinct in aetiology and clinical implications. Additionally, the study combined hazard ratios (HRs) with ORs, which is methodologically inappropriate—a similar issue also occurred in the prior meta-analysis [3] supporting the ISPD suggestion.

We conducted an updated systematic review with more robust methodological approach. Detailed descriptions of the search strategy and statistical method are provided in Supplementary data, Fig. S1, and Tables S1 and S2. We included eight studies [S3–S10] with 25 663 PD patients, adopting the random effect model and Hartung–Knapp–Sidik–Jonkman method for more robust estimation. Study characteristics are summarized in Supplementary data, Table S3. We observed a non-significant trend toward an increased risk of overall PD-associated peritonitis (OR 1.75, 95% CI 0.94–3.28) and enteric peritonitis (OR 1.91, 95% CI 0.72–5.09) with GAS use. Similar results were obtained when pooling HRs (Supplementary data, Fig. S2). Further analysis of proton pump inhibitors (PPIs) or H2RAs exposure versus non-exposure also showed non-significant trends, highlighting the limited evidence linking GAS use to peritonitis (Fig. 1). After adjusting for potential small-study bias using the trim-and-fill method, the associations of GAS, PPIs and H2RAs with both overall and enteric peritonitis remained consistent with the primary analysis (Supplementary data, Table S4). The risk of bias was assessed using the Newcastle-Ottawa Scale (Supplementary data, Tables S5 and S6).

**Figure 1:**
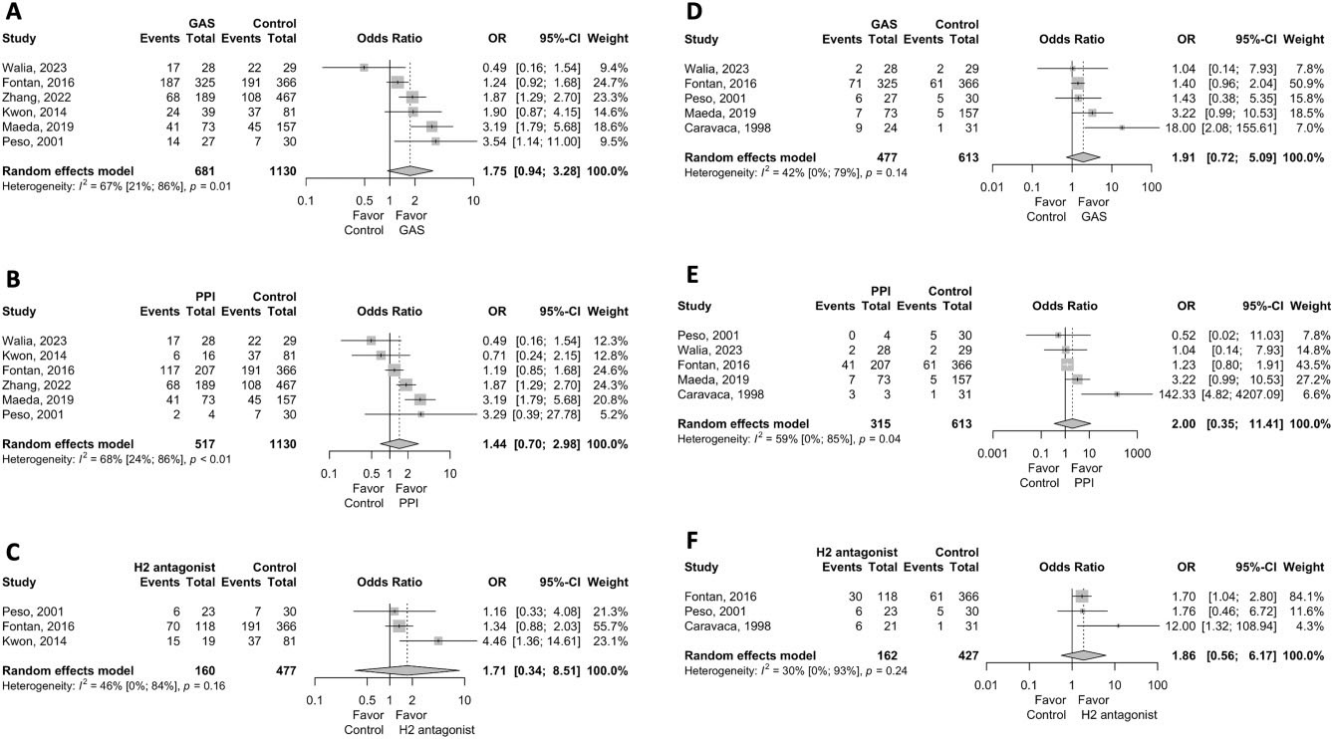
Forest plots for pooled ORs of PD-associated peritonitis development. (**A**) Overall PD-associated peritonitis and the use of GAS. (**B**) Overall PD-associated peritonitis and the use of PPIs. (**C**) Overall PD-associated peritonitis and the use of H2RAs. (**D**) Enteric peritonitis and the use of GAS. (**E**) Enteric peritonitis and the use of PPIs. (**F**) Enteric peritonitis and the use of H2RAs.

Notably, our analysis incorporated data from the Peritoneal Dialysis Outcomes and Practice Patterns Study (PDOPPS), a multinational, multicentre prospective cohort study published in 2024 [S10]. It is surprising that Yao *et al*. did not include this pivotal study, which provides valuable information.

Chronic PPI use, on the other hand, has been linked to risks such as pneumonia, fractures and osteoporosis, even in dialysis patients [S11–S14]. Importantly, the latter complications, especially fractures, have not been observed with H2RAs [S15]. These findings underscore an intriguing discrepancy: although PPIs are generally associated with a higher incidence of adverse effects compared with H2RAs, this trend does not appear to extend to the risk of peritonitis. Furthermore, it is crucial to carefully weigh the benefits and risks beyond peritonitis when prescribing GAS agents.

In conclusion, while the ISPD guidelines and prior studies emphasize risks with H2RAs, our findings suggest these risks may be overestimated. A balanced, evidence-based approach to GAS use in PD patients is essential, considering both potential benefits and risks.

## Supplementary Material

sfaf054_Supplemental_File
