# Supplementary material for: The impact of gastric acid suppressants on peritonitis risk in peritoneal dialysis patients: a systematic review and meta-analysis
Source: Clin Kidney J. 2025 Feb 20;18(3):sfaf054. doi: 10.1093/ckj/sfaf054 (PMC11926588; doi:10.1093/ckj/sfaf054)
Supplement: sfaf054_Supplemental_File [file sfaf054_supplemental_file.pdf]

Supplementary Document

Literature Search Strategy

The current study was conducted in accordance with the Preferred Reporting Items for Systematic Reviews and Meta-Analyses (PRISMA) guidelines and the protocol was registered in PROSPERO (CRD: 42024591771). Two independent investigators (W.Y.H. and C.C.H.) separately conducted searches for studies published before March 30th, 2024 in the databases of PubMed, MEDLINE, the Cochrane Library, and the Embase, without language restrictions. The search strategies focused on identifying published articles that compared the risk of peritonitis between GAS users and non-users. Additionally, we screened relevant trials and reviewed the references of pertinent review articles.

Study Eligibility and Excluding Criteria

Studies were included if they met the following criteria:

1. Enrolled adult peritoneal dialysis patients.
2. Classified patients into GAS (including PPI, H2RA, or others) users and non-user groups.
3. Reported at least one outcome of interest: either overall PD-associated peritonitis or enteric peritonitis (reported as crude events or hazard ratios).

The titles and abstracts of references identified through the search process were initially screened independently by two investigators (W.Y.H. and J.J.C.) to exclude clearly irrelevant studies. Full-text articles of potentially relevant studies were then obtained to assess eligibility. A third investigator (C.C.H.) was consulted to resolve any disagreements regarding eligibility and study categorization.

Data Extraction

Two investigators (W.Y.H. and C.C.H.) independently extracted relevant information from each selected study. Extracted data included study characteristics, participant demographics (age, gender, country), study design, detailed information on interventions, and outcomes of interests (e.g., events of peritonitis, hazard ratios for peritonitis). A third investigator (J.J.C.) was consulted to resolve any disagreements during the data extraction process.

Outcome Measures & Statistical Analysis

The primary outcome was overall PD-associated peritonitis and the secondary outcome was enteric peritonitis. For studies where the event rates of peritonitis in both groups could be extracted, odds ratios were used to pool the effect. For studies reporting only hazard ratios, the

HR and its 95% confidence interval were extracted, and the pooled effect of drug exposure was expressed as a hazard ratio.

Given the inclusion of different study designs (case-control and cohort studies), variations in the definition of drug exposure, and the use of different types of gastric acid suppressants, a random-effects model was deemed more appropriate than a fixed-effects model. For binary outcomes, due to the limited number of included studies and small sample sizes, the Hartung-Knapp-Sidik-Jonkman method was used to estimate between-study variance and confidence intervals, instead of the traditional DerSimonian and Laird approach. For hazard ratios, the generic inverse variance method was employed to pool the effects.

Heterogeneity was assessed using the I^2 statistic and Cochran's Q test (with a p-value < 0.1 indicating heterogeneity). Considering potential publication bias due to the predominance of published retrospective cohort studies, sensitivity analysis was conducted using the trim-and-fill method. All analyses were performed using the *metabin & metagen* functions in the meta package in R (version 4.0.2).

Risk of Bias Assessments

The risk of bias for the included studies was evaluated using the Newcastle-Ottawa Scale. Two independent reviewers (W.Y.H. and J.J.C.) assessed the risk of bias across each domain. Any disagreements between the reviewers were resolved through discussion with a third author (C.C.H.).

Supplementary Table Legend

Supplementary Table 1. Search strategy for each database
Supplementary Table 2. Reasons for excluding studies
Supplementary Table 3. Characteristic of enrolled studies
Supplementary Table 4: Sensitivity analysis by Trim & Fill Method
Supplementary Table 5: Risk of bias assessment by Newcastle-Ottawa Scale (case-controlled studies)
Supplementary Table 6: Risk of bias assessment by Newcastle-Ottawa Scale (cohort study)
PRISMA checklist

Supplementary Figure Legend

Supplementary Figure 1. PRISMA 2020 flow diagram
Supplementary Figure 2. Forest plot for pooled hazard ratio of peritonitis development and the use of gastric acid suppressants (A), Proton Pump Inhibitor (B) and H2 blocker (C)

Supplementary Table 1. Search strategy for each database

Pubmed through March 30th, 2024

#1 (peritoneal dialysis) AND ((((((proton pump inhibitors) OR (H2 receptor antagonists)) OR (H2 receptor blockers)) OR (gastric acid inhibitor)) OR (acid suppressive therapy)) OR (anti-ulcer agents))

Result: 145

EMbase through March 30th, 2024

'peritoneal dialysis'/exp AND ('proton pump inhibitor'/exp OR 'histamine h2 receptor antagonist'/exp OR 'gastric acid inhibitor':ti,ab OR 'acid suppressive therapy'/mj OR 'antiulcer agent'/mj)

Result: 345

Medline through March 30th, 2024

#1 Peritoneal dialysis.mp. or Peritoneal Dialysis/
#2 proton pump inhibitors.mp. or Proton Pump Inhibitors/
#3 Histamine H2 Antagonists/ or H2 receptor antagonists.mp.
#4 gastric acid inhibitor.mp.
#5 acid suppressive therapy.mp.
#6 anti ulcer agents.mp. or Anti-Ulcer Agents/
#7 2 or 3 or 4 or 5 or 6
#8 1 and 7

Result: 34

Cochrane library through March 30th, 2024

#1 MeSH descriptor: [Peritoneal Dialysis] explode all trees
#2 (peritoneal dialysis)
#3 MeSH descriptor: [Proton Pump Inhibitors] explode all trees
#4 (Proton Pump Inhibitors)
#5 MeSH descriptor: [Histamine H2 Antagonists] explode all trees
#6 (Histamine H2 Antagonists)
#7 (gastric acid inhibitors)
#8 (acid suppressive therapy)
#9 MeSH descriptor: [Anti-Ulcer Agents] explode all trees
#10 (Anti-Ulcer Agents)
#11 #1 or #2
#12 #3 or #4 or #5 or #6 or #7 or #8 or #9 or #10
#13 #11 and #12

Result 8

Supplementary Table 2. Reasons for excluding studies

**Author, year**	**Title**	**Reason for exclusion**	**Result**
1.Gabella, 1998	Therapy with Gastric Acid Inhibitors Is Not Related to Enteric Peritonitis in Peritoneal Dialysis	No result of interest	Exclusion
2.Diskin, 2008	Immunology and the evaluation of risk factors for development of spontaneous bacterial peritonitis	No result of interest	Exclusion
3.Choi, 2008	Are gastric acid suppressants a risk factor for enteric peritonitis?	No result of interest	Exclusion
4.Nessim, 2008	Gastric acid suppression and the risk of enteric peritonitis in peritoneal dialysis patients	No result of interest	Exclusion
5. Chan, 2021	Risk of peritonitis after gastroscopy in peritoneal dialysis patients	No result of interest	Exclusion

Supplementary Table 3. Characteristic of enrolled studies

No	Author, Year	Country	Study design	N	Age	Male (%)	Definition of PD-associated peritonitis	GAS used (n)	PD vintage (months)	PD modality (%)	Brief conclusion
1	Caravaca 1998	Spain	Single center, Retrospective case control study	55	53.3	21 (38.1)	Only enteric peritonitis denifed[a]	Omeprazole (3) H2RA (24)	24.7 ± 15.0	CAPD (100)	Positive association between GAS and enteric peritonitis
2	Peso 2001	Spain	Single center, Retrospective case control study	57	50.9	29 (50.8)	Only enteric peritonitis denifed[b]	Omeprazole (4) H2RA (23)	33.4 ± 42	CAPD (56) APD (44)	No association between GAS and enteric peritonitis
3	Kwon 2014	Korea	Single center, Retrospective case control study	120	49 - 51	53 (53.5)	By ISPD guideline[c]	PPI (16) H2RA (19) Others (7)	N/A	CAPD (81.6) APD (18.4)	Positive association between H2RAs and PD-associated peritonitis
4	Fontan 2016	Spain	Single center, Retrospective cohort study	691	58.9	425 (61.4)	N/A	PPI (207) H2RA (118)	169	CAPD (62.5) APD (37.5)	Positive association between GAS and enteric peritonitis
5	Maeda 2019	Japan	Single center, Retrospective cohort study	230	64.0	165 (71.7)	By ISPD guideline[c]	PPI (73)	N/A	N/A	Positive association between PPIs and PD-associated peritonitis
6	Zhang 2022	China	Two centers, Retrospective cohort study	656	53.0	367 (55.9)	By ISPD guideline[c]	PPI (189)	N/A	N/A	Positive association between PPIs and PD-associated peritonitis
7	Walia 2023	Australia	Single center, Retrospective cohort study	57	65	39 (68.4)	By ISPD guideline[c]	PPI (28)	N/A	N/A	No association between PPIs and PD-associated peritonitis
8	Goldman 2024	Eight countries	Multicenter, Prospective cohort study	23797	59.4	13597 (56.7)	By facility-reported diagnosis	PPI (6020) H2RA (1382)	5.4 ± 4.5	APD (72.4) Assisted PD (27.4)	No association between GAS and PD-associated peritonitis

Abbreviations:

APD, automated peritoneal dialysis; CAPD, continuous ambulatory peritoneal dialysis; GAS, gastric acid suppressants; H2RA, histamine H2-receptor antagonists; ISPD, International Society for Peritoneal Dialysis; N/A, not available; PD, peritoneal dialysis; PPI, proton pump inhibitors

Footnotes:

[a] Intestinal flora incubated, excluding polymicorbial infection, exit infection, recent genital, intestinal, or urinary tract instrumentation

[b] Intestinal flora incubated, excluding exit infection and genitourinary infection

[c] Fulfill at least two of the following diagnostic criteria: (1) Abdominal pain or cloudy PD effluent (2) leukocytosis in the peritoneal fluid effluent (white blood cell > $100/mm^3$, with at least 50% polymorphonuclear leukocytes) (3) A positive Gram stain or positive culture from PD effluent

Supplementary Table 4: Sensitivity analysis by Trim & Fill Method

	GAS	PPI	H2 blocker
All peritonitis	OR 1.62<br>(95% CI 0.90-2.90)	OR 1.66<br>(95% CI 0.80-3.48)	OR 1.71<br>(95% CI 0.34-8.51)
Enteric peritonitis	OR 1.62<br>(95% CI 0.39-6.68)	OR 2.00<br>(95% CI 0.35-11.41)	OR 1.86<br>(95% CI 0.56-6.17)

Supplementary Table 5: Risk of bias assessment by Newcastle-Ottawa Scale (case-controlled studies)

GAS exposure and peritonitis development: case control studies										
	SELECTION				COMPARABILITY	OUTCOME			NOS score	Risk of bias
Study	Representative ness of the exposed cohort (1)	Selection of the non-exposed cohort (1)	Ascertainment of exposure (1)	Demonstration that outcome of interest was not present at start of study (1)	Comparability of cohorts on the basis of the design or analysis (2)	Assessment of outcome (1)	Was follow-up long enough for outcomes to occur (1)	Adequacy of follow up of cohorts (1)		
Caravaca, 1998	1	1	1	1	0[a]	0[b]	1	1	**6**	**M**
Peso, 2001	1	1	1	1	0[a]	0[b]	1	1	**6**	**M**
Kwon, 2014	1	1	1	1	0[a]	1	1	1	**7**	**L**

Footnote: A study can be awarded a maximum of one point for each item within the Selection and Outcome categories if it is high quality. A maximum of two points can be given for Comparability. The Newcastle-Ottawa scale have total 8 items within 3 domain and the total maximum score is 9. The total score for a study ranges from 0 to 9, with higher scores indicating a lower risk of bias. In addition, the NOS provides guidance on how to interpret the scores, such as categorizing studies with scores of 0–3, 4–6, and 7–9 as having high, moderate, or low risk of bias, respectively

[a] Covariates were not matched between groups.
[b] There was no clear definition of peritonitis.

Supplementary Table 6: Risk of bias assessment by Newcastle-Ottawa Scale (cohort study)

GAS exposure and peritonitis development: cohort studies										
	SELECTION				COMPARABILITY	OUTCOME			NOS score	Risk of bias
Study	Representative ness of the exposed cohort (1)	Selection of the non-exposed cohort (1)	Ascertainment of exposure (1)	Demonstration that outcome of interest was not present at start of study (1)	Comparability of cohorts on the basis of the design or analysis (2)	Assessment of outcome (1)	Was follow-up long enough for outcomes to occur (1)	Adequacy of follow up of cohorts (1)		
Fontan, 2016	1	1	1	1	2	0[a]	1	1	**8**	**L**
Maeda, 2019	1	1	1	1	2	1	1	1	**9**	**L**
Zhang, 2022	1	1	1	1	2	1	1	1	**9**	**L**

Walia, 2023	1	1	1	1	1[b]	1	1	1	**8**	**L**
Goldman,2024	1	1	1	1	2	0[a]	1	1	**8**	**L**

Footnote: A study can be awarded a maximum of one point for each item within the Selection and Outcome categories if it is high quality. A maximum of two points can be given for Comparability. The Newcastle-Ottawa scale have total 8 items within 3 domain and the total maximum score is 9. The total score for a study ranges from 0 to 9, with higher scores indicating a lower risk of bias. In addition, the NOS provides guidance on how to interpret the scores, such as categorizing studies with scores of 0–3, 4–6, and 7–9 as having high, moderate, or low risk of bias, respectively

[a] There was no clear definition of peritonitis.
[b] The age and gender were not adjusted between groups.

Supplementary Figure 1. PRISMA 2020 flow diagram

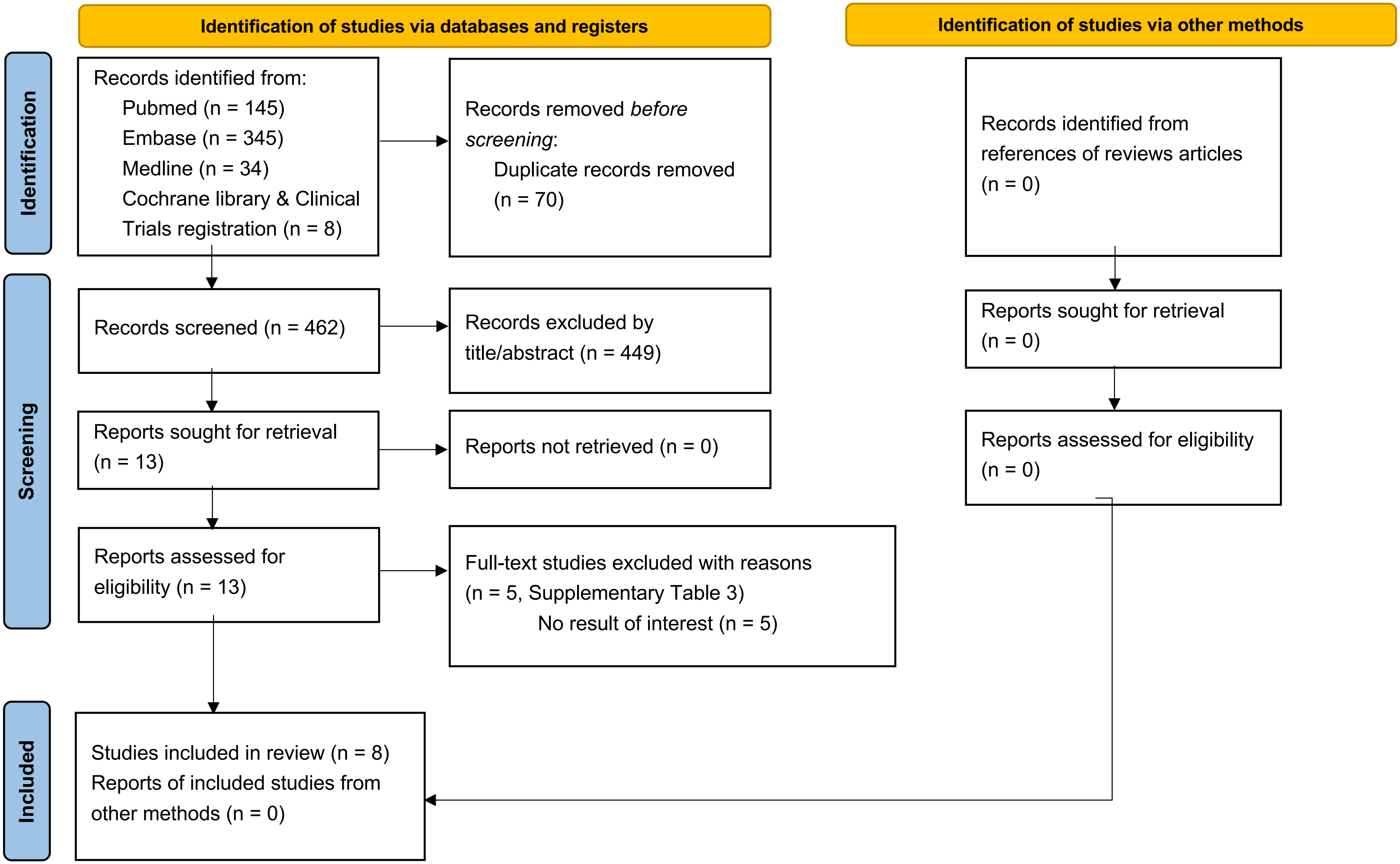

Supplementary Figure 2. Forest plot for pooled hazard ratio of peritonitis development and the use of gastric acid suppressants (A), Proton Pump Inhibitor (B) and H2 blocker (C)

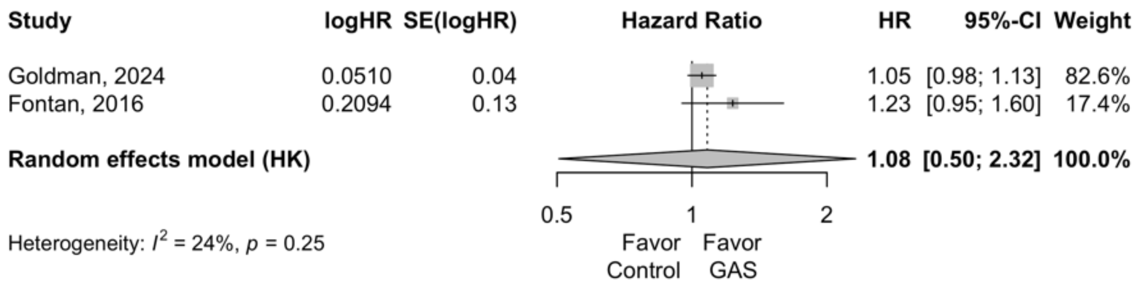

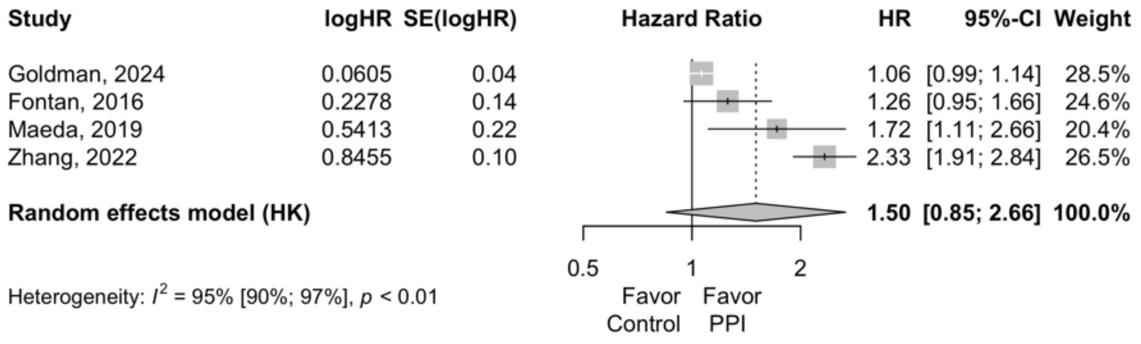

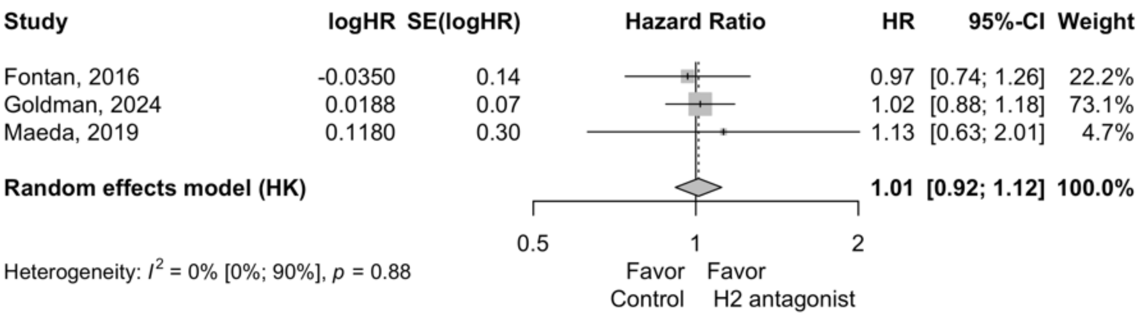

Supplementary Reference

S1. Gabella P, Singhal M, Szabo T, Vas S, Oreopoulos DG; Therapy with Gastric Acid Inhibitors is not Related to Enteric Peritonitis in Peritoneal Dialysis. Peritoneal Dialysis International: Journal of the International Society for Peritoneal Dialysis 1998; 18(6):656-657.

S2. Nessim SJ, Tomlinson G, Bargman JM, Jassal SV; Gastric Acid Suppression and the Risk of Enteric Peritonitis in Peritoneal Dialysis Patients. Peritoneal Dialysis International: Journal of the International Society for Peritoneal Dialysis 2008; 28(3):246-251.

S3. Caravaca F, Ruiz-Calero R, Dominguez C; Risk factors for developing peritonitis caused by micro-organisms of enteral origin in peritoneal dialysis patients. Perit Dial Int 1998; 18(1):41-5.

S4. Del Peso G, Bajo MA, Gadola L, et al.; Diverticular Disease and Treatment with Gastric Acid Inhibitors do not Predispose to Peritonitis of Enteric Origin in Peritoneal Dialysis Patients. Peritoneal Dialysis International: Journal of the International Society for Peritoneal Dialysis 2001; 21(4):360-364.

S5. Kwon JE, Koh SJ, Chun J, et al.; Effect of gastric acid suppressants and prokinetics on peritoneal dialysis-related peritonitis. World J Gastroenterol 2014; 20(25):8187-94.

S6. Perez-Fontan M, Machado Lopes D, Garcia Enriquez A, et al.; Inhibition of Gastric Acid Secretion by H2 Receptor Antagonists Associates a Definite Risk of Enteric Peritonitis and Infectious Mortality in Patients Treated with Peritoneal Dialysis. PLoS One 2016; 11(2):e0148806.

S7. Maeda S, Yamaguchi M, Maeda K, et al.; Proton pump inhibitor use increases the risk of peritonitis in peritoneal dialysis patients. PLoS One 2019; 14(11):e0224859.

S8. Zhang Y, Li J, Chen Z, et al.; Proton pump inhibitor usage associates with higher risk of first episodes of pneumonia and peritonitis in peritoneal dialysis patients. Ren Fail 2022; 44(1):1623-1631.

S9. Walia N, Rao N, Garrett M, Yates K, Malone S, Holmes C; Proton pump inhibitor use and the risk of peritoneal dialysis associated peritonitis. Intern Med J 2023; 53(3):397-403.

S10. Goldman S, Zhao J, Bieber B, et al.; Gastric Acid Suppression Therapy and Its Association with Peritoneal Dialysis-Associated Peritonitis in the Peritoneal Dialysis Outcomes and Practice Patterns Study (PDOPPS). Kidney360 2024; 5(3):370-379.

S11. Alanazi AS, Almutairi H, Gupta JK, et al.; Osseous implications of proton pump inhibitor therapy: An umbrella review. Bone Rep 2024; 20:101741.

S12. Khalili H, Huang ES, Jacobson BC, Camargo CA, Jr., Feskanich D, Chan AT; Use of proton pump inhibitors and risk of hip fracture in relation to dietary and lifestyle factors: a prospective cohort study. BMJ 2012; 344:e372.

S13. Fusaro M, D'Arrigo G, Pitino A, et al.; Increased Risk of Bone Fractures in Hemodialysis Patients Treated with Proton Pump Inhibitors in Real World: Results from the Dialysis Outcomes and Practice Patterns Study (DOPPS). J Bone Miner Res 2019; 34(12):2238-2245.

S14. Zhang Y, Deng D, Zhang R, Yi J, Dong J, Sha L; Relationship between Proton Pump Inhibitors and Adverse Effects in Hemodialysis Patients: A Systematic Review and Meta-Analysis. Kidney Blood Press Res 2022; 47(9):545-555.

S15. Vangala C, Niu J, Lenihan CR, Mitch WE, Navaneethan SD, Winkelmayer WC; Proton Pump Inhibitors, Histamine-2 Receptor Antagonists, and Hip Fracture Risk among Patients on Hemodialysis. Clin J Am Soc Nephrol 2018; 13(10):1534-1541.
